# The gender dependent influence of sodium bicarbonate supplementation on anaerobic power and specific performance in female and male wrestlers

**DOI:** 10.1038/s41598-020-57590-x

**Published:** 2020-02-05

**Authors:** Krzysztof Durkalec–Michalski, Emilia E. Zawieja, Bogna E. Zawieja, Patrycja Michałowska, Tomasz Podgórski

**Affiliations:** 1 Institute of Human Nutrition and Dietetics, Poznań University of Life Sciences, Poznań, 60-624 Poland; 2 Department of Food and Nutrition, Poznań University of Physical Education, Poznań, 61-871 Poland; 3 Department of Mathematical and Statistical Methods, Poznań University of Life Sciences, Poznań, 60-637 Poland; 4 Department od Physiology and Biochemistry, Poznań University of Physical Education, Poznań, 61-871 Poland

**Keywords:** Physiology, Medical research

## Abstract

The aim of this study was the assessment of progressive low-dose sodium bicarbonate (NaHCO_3_) supplementation on the anaerobic indices in two bouts of Wingate tests (WT) separated by wrestling-specific performance test and assessing the gender differences in response. Fifty-one (18 F) wrestlers completed a randomized trial of either a NaHCO_3_ (up to 100 mg·kg^−1^) or a placebo for 10 days. Before and after treatment, athletes completed an exercise protocol that comprised, in sequence, the first WT_1_, dummy throw test (DT), and second WT_2_. The number of completed throws increased significantly in males from 19.3 ± 2.6 NaHCO_3pre_ to 21.7 ± 2.9 NaHCO_3post_. ΔWT_2_-WT_1_ improved particularly in the midsection of 30-s WT on NaHCO_3_. However, no significant differences were found in peak power (PP), power drop (PD) and average power (AP) (analyzed separately for each WT), and ΔWT_2_-WT_1_ in PP and PD. Interaction with gender was significant for AP, PP and PD, every second of WT_1_ and WT_2_, as well as DT test. In conclusion, our study suggests that the response to NaHCO_3_ may be gender-specific and progressive low-dose NaHCO_3_ supplementation allows the advantageous strengthening of wrestling-specific performance in males. It can also lead to maintenance of high anaerobic power mainly in the midsection of the 30-s Wingate test.

## Introduction

Dietary supplement use is greater in athletes than in the general population^1^. Some supplements, when ingested properly, can improve the athlete’s health and performance, but others are taken even though they have no proved influence. One of the supplements whose effectiveness is indicated in International Olympic Committee (IOC) as well as International Society of Sports Nutrition statements, especially for high-performance athletes, is sodium bicarbonate (NaHCO_3_)^2,3^. NaHCO_3_ supplementation increases extracellular bicarbonate concentration, which causes blood alkalosis^4^. Because of the greater pH gradient between the muscle cells and extracellular fluids, H^+^ produced during exercise are transported more easily leads to greater efflux of H^+^ and La^−^ from the exercising muscle^3–5^. This is particularly important because intramuscular acidosis can cause muscular fatigue based on different mechanisms: (1) impaired glycolysis because of reduced activity of key enzymes such as glycogen phosphorylase and phosphofructokinase; (2) hindered muscle’s contraction capacity due to the competition of H^+^ with calcium ions; (3) inhibition of oxidative phosphorylation; (4) compromised resynthesis of phosphocreatine at low pH^6^. The increase in buffer capacity by NaHCO_3_ supplementation can therefore allow to sustain muscle contractility during intense exercise and delay muscle fatigue^5,6^.

Improvements in performance contributed to increases in buffering capacity are likely confined to short and high-intensity tasks which can be limited by acid-base disturbances and combat sports are one of them. Wrestling is a high-intensity competitive sport discipline, in which glycolysis is a substantial energy system. For instance, blood lactate concentrations rise up to ~12.5 mmol·L^−1^ after a simulated wrestling combat^7^. The anaerobic power is crucial to perform wrestling attacks, to lift and/or throw an opponent during offensive actions in wrestling, as well as to resist the opponent’s attacks^8^. Thus, wrestling athletes would benefit from NaHCO_3_ notably. However, studies on NaHCO_3_ supplementation in wrestling are scarce. In judo, NaHCO_3_ caused a significant improvement in judo-specific performance, confirming its efficacy in intermittent supramaximal specific to discipline bouts of exercises, in which fatigue is evident^9^. The *Special Judo Fitness Test* (SJFT) result was improved in 0.3 g·kg^−1^ NaHCO_3_ than in placebo (PLA) treatment^9^. Furthermore, in striking disciplines such as boxing or taekwondo, the same dose of NaHCO_3_ increased exercise capacity related to the total punch efficacy^10^ or the attack time of a simulated taekwondo combat^11^, respectively. In contrary, in a study by Felippe *et al*.^12^ only combined treatment with NaHCO_3_ and caffeine resulted in significant increases in judo performance compared with PLA. In a previous study by our lab, a dose of up to 0.1 g·kg^−1^ NaHCO_3_ had no effect on wrestling performance in specific dummy throw test (DT) compared to PLA^13^. However, even though the doses used in the study seemed to be too small to improve the power in two Wingate tests (WT) and the number of wrestling *suplex* throws, the time-to-peak power decreased significantly with NaHCO_3_, but only in the second WT^13^.

It is therefore possible that NaHCO_3_ supplementation effect is more pronounce later in the multiple-bout workout when the capacity of dealing with H^+^ is significantly exhausted. For instance, in a study by Artioli *et al*.^9^ the improvement in peak and average power was observed only in the two final bouts of four 30-s upper body WTs^9^. Similarly, Tobias *et al*.^14^ observed that NaHCO_3_ improved mean power only in forth bout of 30-s upper WT by ~9% and ~14%, respectively. Olivera *et al*.^15^ also noted an increase in total mechanical work done in the last two bouts (bouts 3 + 4: + 5.93%, *p* = 0.02) of four 30-s sprints.

Since it seems that sodium bicarbonate may influence the latter bouts of high intensity exercise, we aimed at assessing the gender-related influence of progressive-dose NaHCO_3_ loading regimen on the difference in power between two bouts of Wingate tests separated by wrestling-specific exercise performance test, simulating combat during an intense competition round. We analyzed both genders separately since it seems possible that females respond to sodium bicarbonate to a lesser extent than males. That can result from the differences in muscle anatomy and physiology. On one hand men are usually stronger and more powerful, and on the other hand women are less fatigable^16^. Moreover, females have smaller type II fibers than men^17,18^, while males have greater glycolytic capacity^19,20^. Finally, the exercise-induced pH drop is also greater in males^20^. Therefore, we hypothesized that NaHCO_3_ supplementation in men will have a greater beneficial effect on muscle power and throwing performance.

## Results

### Wingate anaerobic power indices

The interaction with gender was significant for average power (AP) (*p* < 0.0001), power drop (PD) (*p* < 0.0001), and peak power (PP) (*p* < 0.0001). However, no significant differences in AP, PD and PP were found after NaHCO_3_ and PLA interventions neither in females nor in males (Table 1). There were no significant gender interactions for the differences between WT_2_ and WT_1_ (Δ WT_2_-WT_1_) in AP, PD and PP. Δ WT_2_-WT_1_ in AP, PP and PD were not significantly affected by NaHCO_3_ and PLA treatments in both genders (Table 2).

**Table 1:** Power characteristics before and after supplementation in female and male wrestlers.

			Peak Power		Power Drop		Average Power	
			(W)	(W∙kg^−1^)	(W)	(W∙kg^−1^)	(W)	(W∙kg^−1^)
Females	NaHCO_3pre_	WT_1_	497 ± 100	9.3 ± 1.2	268 ± 65	5.0 ± 0.9	339 ± 59	6.3 ± 0.7
		WT_2_	465 ± 100	8.7 ± 1.1	265 ± 67	5.0 ± 1.1	316 ± 66	5.8 ± 0.8
	NaHCO_3post_	WT_1_	529 ± 93	9.9 ± 0.9	317 ± 66	5.9 ± 0.9	338 ± 55	6.3 ± 0.5
		WT_2_	532 ± 113	9.8 ± 1.1	319 ± 68	6.0 ± 0.9	336 ± 62	6.2 ± 0.7
	PLA_pre_	WT_1_	622 ± 107	9.9 ± 1.9	354 ± 105	5.6 ± 1.7	413 ± 48	6.4 ± 0.7
		WT_2_	591 ± 110	9.4 ± 1.8	314 ± 122	5.0 ± 2.1	416 ± 45	6.5 ± 0.5
	PLA_post_	WT_1_	646 ± 112	10.2 ± 1.8	379 ± 105	6.0 ± 1.8	421 ± 41	6.6 ± 0.4
		WT_2_	627 ± 121	9.9 ± 1.8	359 ± 97	5.6 ± 1.5	406 ± 57	6.3 ± 0.6
Males	NaHCO_3pre_	WT_1_	1023 ± 265	13.1 ± 2.5	640 ± 212	8.2 ± 2.3	589 ± 84	7.5 ± 0.5
		WT_2_	965 ± 250	12.3 ± 2.4	614 ± 196	7.8 ± 1.9	556 ± 96	7.0 ± 0.8
	NaHCO_3post_	WT_1_	1089 ± 244	14.0 ± 2.4	704 ± 194	9.0 ± 2.2	592 ± 92	7.5 ± 0.6
		WT_2_	1067 ± 228	13.7 ± 2.0	723 ± 173	9.3 ± 2.0	578 ± 97	7.3 ± 0.7
	PLA_pre_	WT_1_	954 ± 322	12.6 ± 3.1	608 ± 255	8.0 ± 2.8	564 ± 131	7.5 ± 0.9
		WT_2_	897 ± 270	11.8 ± 2.8	584 ± 194	7.7 ± 2.2	540 ± 126	7.1 ± 1.0
	PLA_post_	WT_1_	990 ± 267	13.1 ± 2.4	646 ± 193	8.6 ± 2.0	569 ± 128	7.5 ± 0.8
		WT_2_	935 ± 238	12.5 ± 2.4	629 ± 173	8.4 ± 2.0	542 ± 128	7.2 ± 1.0

Data are presented at mean ± SD. NaHCO_3pre_, before sodium bicarbonate supplementation; NaHCO_3post_, after sodium bicarbonate supplementation; PLA_pre_, before placebo; PLA_post_, after placebo; WT_1_, the first Wingate test; WT_2_, the second Wingate test.

**Table 2:** The difference in power between WT_2_ and WT_1_ (Δ WT_2_-WT_1_) before and after supplementation.

		Peak Power		Power Drop		Average Power	
		(W)	(W∙kg^−1^)	(W)	(W∙kg^−1^)	(W)	(W∙kg^-1^)
Females	NaHCO_3pre_	−32.5 ± 57.1	−0.6 ± 1.1	−3.0 ± 48.1	0.0 ± 0.9	−22.6 ± 39.6	−0.4 ± 0.8
	NaHCO_3post_	2.6 ± 28.2	−0.1 ± 0.4	2.3 ± 24.5	0.0 ± 0.5	−1.5 ± 17.4	0.0 ± 0.3
	PLA_pre_	−30.7 ± 22.5	−0.5 ± 0.3	−40.7 ± 41.4	−0.6 ± 0.6	3.8 ± 11.6	0.1 ± 0.2
	PLA_post_	−18.4 ± 55.2	−0.3 ± 0.9	−19.8 ± 52.2	−0.4 ± 0.8	−14.6 ± 23.9	−0.2 ± 0.4
Males	NaHCO_3pre_	−57.6 ± 106.3	−0.8 ± 1.4	−26.1 ± 108.3	−0.4 ± 1.4	−32.7 ± 42.8	−0.4 ± 0.6
	NaHCO_3post_	−22.6 ± 103.8	−0.3 ± 1.3	18.4 ± 122.4	0.3 ± 1.6	−14.5 ± 28.6	−0.2 ± 0.4
	PLA_pre_	−57.8 ± 120.5	−0.8 ± 1.5	−24.0 ± 129.6	−0.4 ± 1.7	−23.3 ± 60.3	−0.3 ± 0.8
	PLA_post_	−55.6 ± 58.8	−0.7 ± 0.8	−17.7 ± 78.7	−0.1 ± 1.1	−26.6 ± 28.6	−0.3 ± 0.4

Data are presented at mean ± SD. NaHCO_3pre_, before sodium bicarbonate supplementation; NaHCO_3post_, after sodium bicarbonate supplementation; PLA_pre_, before placebo; PLA_post_, after placebo.

The interactions between treatment x period as regards power were significant in seconds 12 (*p* = 0.0106) and 16 (*p* = 0.0398) in all wrestlers. Gender interaction was significant in each second of WT. Moreover, gender x treatment interaction was significant in seconds: 10 (*p* = 0.0343), 11 (*p* = 0.0438), 12 (*p* = 0.0153), 15 (*p* = 0.0461), 16 (*p* = 0.0365), 17 (*p* = 0.0280), 21 (*p* = 0.0248), 23 (*p* = 0.0377), 26 (*p* = 0.0474), 28 (*p* = 0.0304), 29 (*p* = 0.0181) and 30 (*p* = 0.0359). In females significant changes were observed in seconds: 1 (*p* = 0.0204), 12 (*p* = 0.0180), 21 (*p* = 0.0070), 25 (*p* = 0.0343), 28 (*p* = 0.0083), 29 (*p* = 0.0294) and 30 (*p* = 0.0463). In males in seconds: 12 (*p* = 0.0269), 16 (*p* = 0.0409) and 17 (*p* = 0.0082).

**Figure 1:**
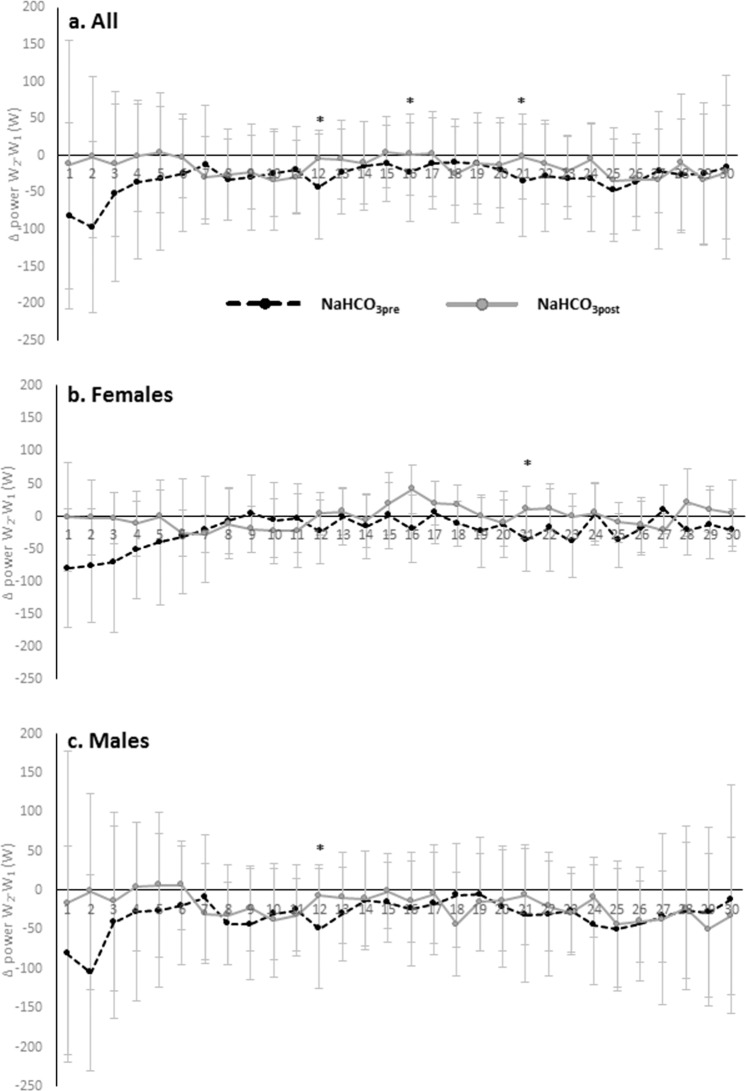
Difference in power indices between WT_2_ and WT_1_ before and after NaHCO_3_ supplementation. (**a**) In all participants, (**b**) in females, (**c**) in males. Data are presented at mean ± SD. *NaHCO_3post_ significantly different from NaHCO_3pre_.

**Figure 2:**
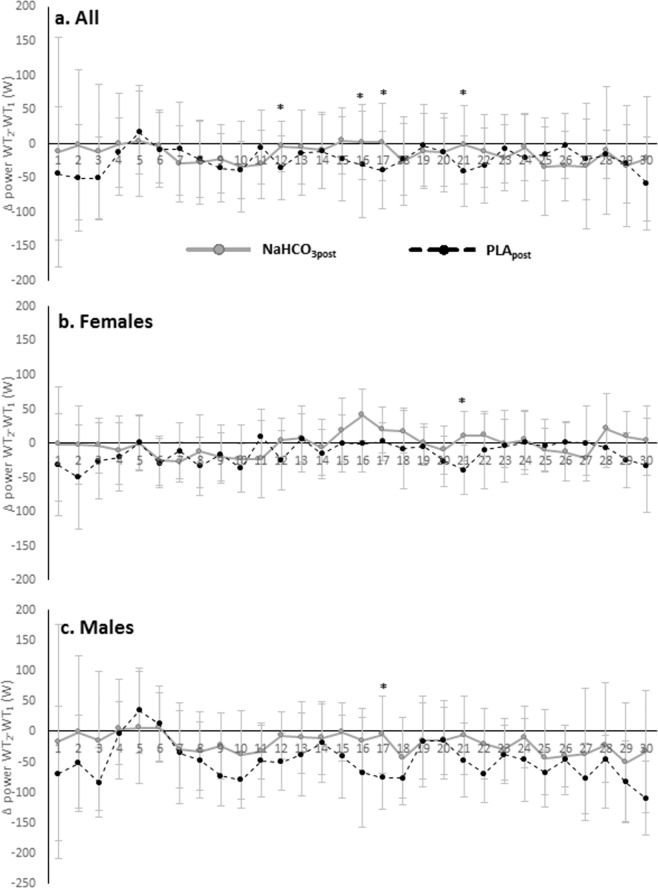
Difference in power indices between WT_2_ and WT_1_ (Δ WT_2_-WT_1_) in NaHCO_3post_ vs PLA_post_ (**a**) in all participants, (**b**) in females, (**c**) in males. Data are presented at mean ± SD. *NaHCO_3post_ significantly different from PLA_post_.

In all participants the difference in power indices between WT_2_ and WT_1_ (Δ power WT_2_-WT_1_) improved significantly NaHCO_3post_ vs NaHCO_3pre_ in seconds: 12 (*p* = 0.0413), 16 (*p* = 0.0199) and 21 (*p* = 0.0430) (Fig. 1a). Furthermore, Δ power WT_2_-WT_1_ NaHCO_3post_ was significantly lower than PLA_post_ in seconds: 12 (*p* = 0.0144), 16 (*p* = 0.0370), 17 (*p* = 0.0125) and 21 (*p* = 0.0166) (Fig. 2a). In second 12 Δ power WT_2_-WT_1_ decreased significantly on PLA (PLA_post_ vs PLA_pre_) (*p* = 0.0368). The gender interactions were recorded in seconds: 13 (*p* = 0.0382), 17 (*p* = 0.0174), 18 (*p* = 0.0187), 22 (*p* = 0.0428), 24 (*p* = 0.0082), 25 (*p* = 0.0149), 26 (*p* = 0.0144), 27 (*p* = 0.0123), 28 (*p* = 0.0336) and 29 (*p* = 0.0349), respectively. In females Δ power WT_2_-WT_1_ increased significantly NaHCO_3post_ vs NaHCO_3pre_ only in second 21 (*p* = 0.0475) and was higher NaHCO_3post_ than PLA_post_ (*p* = 0.0130) (Figs. 1b and 2b). In second 12 (*p* = 0.0388), 21 (*p* = 0.0406), 28 (*p* = 0.0294) and 29 (*p* = 0.0279) Δ power WT_2_-WT_1_ decreased significantly on PLA (PLA_post_ vs PLA_pre_). In males Δ power WT_2_-WT_1_ increased significantly NaHCO_3post_ vs NaHCO_3pre_ only in second 12 (*p* = 0.0488) and in second 17 was higher NaHCO_3post_ than PLA_post_ (*p* = 0.0063) (Figs. 1c and 2c). Furthermore, in second 16 (*p* = 0.0316) and 17 (*p* = 0.0045) Δ power WT_2_-WT_1_ decreased significantly on PLA (PLA_post_ vs PLA_pre_).

### Dummy throw test

**Figure 3:**
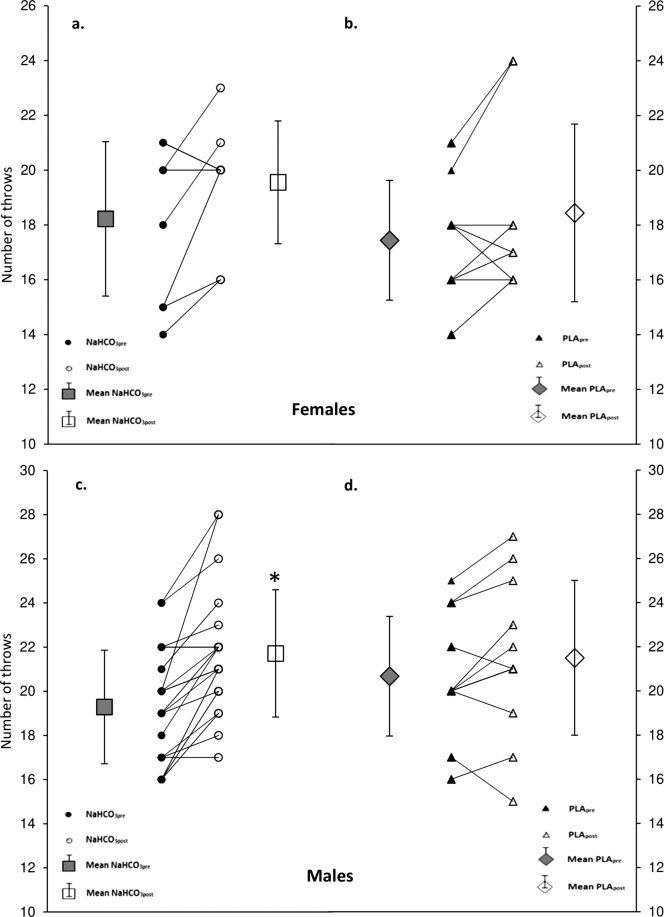
Total number of throws in dummy throw test. (**a**) In females before and after NaHCO_3_, (**b**) in females before and after PLA, (**c**) in males before and after NaHCO_3_, (**d**) in males before and after PLA. Data are presented at mean ± SD, and individual raw data. *NaHCO_3post_ significantly different from NaHCO_3pre_.

The interactions between treatment x period was significant (*p* < 0.0297). In females the number of completed throws was unchanged NaHCO_3pre_ vs NaHCO_3post_ (from 18.2 ± 2.8 to 19.6 ± 2.2 throws, *p* = 0.3766) (Fig. 3a). However in males, it increased significantly by ~12% form 19.3 ± 2.6 to 21.7 ± 2.9 throws (*p* < 0.0001, Fig. 3c). No significant changes were also observed PLA_pre_ vs PLA_post_ (females: *p* = 0.9185; males: *p* = 0.7174) and NaHCO_3post_ vs PLA_post_ (females: *p* = 1.0000; males: *p* = 1.0000) (Fig. 3b,d).

### Blood sample analysis

Before and after supplementation no significant differences in glucose, lactate and pyruvate concentrations were found neither for female nor for male wrestlers (Table 3).

**Table 3:** Glucose, lactate and pyruvate concentrations before and after exercise tests in female and male wrestlers.

		Glucose (mg∙dL^−1^)		Lactate (mmol∙L^−1^)		Pyruvate (mmol∙L^−1^)	
		PRE_exercise_	POST_exercise_	PRE_exercise_	POST_exercise_	PRE_exercise_	POST_exercise_
Females	NaHCO_3pre_	125.1 ± 8.6	137.4 ± 25.0	1.6 ± 0.4	15.9 ± 1.9	0.24 ± 0.05	0.62 ± 0.09
	NaHCO_3post_	104.2 ± 26.2	117.3 ± 28.7	1.6 ± 0.4	14.4 ± 5.4	0.29 ± 0.07	0.53 ± 0.13
	PLA_pre_	122.7 ± 19.7	135.1 ± 26.2	1.8 ± 0.8	14.8 ± 2.2	0.26 ± 0.06	0.62 ± 0.09
	PLA_post_	110.8 ± 20.6	120.6 ± 34.7	1.6 ± 0.8	14.4 ± 3.3	0.25 ± 0.09	0.51 ± 0.11
Males	NaHCO_3pre_	107.0 ± 25.6	123.6 ± 34.0	1.5 ± 0.7	15.9 ± 3.5	0.21 ± 0.06	0.51 ± 0.14
	NaHCO_3post_	113.8 ± 23.4	134.7 ± 29.5	1.5 ± 0.5	16.5 ± 4.0	0.20 ± 0.04	0.54 ± 0.14
	PLA_pre_	124.1 ± 20.7	135.5 ± 22.5	2.1 ± 0.9	17.1 ± 3.0	0.22 ± 0.07	0.52 ± 0.17
	PLA_post_	113.6 ± 9.2	136.0 ± 19.4	2.0 ± 0.8	16.7 ± 2.6	0.22 ± 0.08	0.52 ± 0.05

Data are presented at mean ± standard deviation (SD). NaHCO_3pre_, before sodium bicarbonate supplementation; NaHCO_3post_, after sodium bicarbonate supplementation; PLA_pre_, before placebo; PLA_post_, after placebo.

## Discussion

In this study we showed that progressive supplementation of up to 100 mg·kg^−1^ sodium bicarbonate did not significantly influence AP, PD and PP characteristics in two Wingate tests. However, it improved power maintenance in the midsection of the 30-s Wingate test and performance in wrestling-specific dummy throw test. We observed that gender was a significant factor potentially influencing the effectiveness of such a treatment. Gender interaction was significant for AP, PD and PP, but possibly the dose was too small to elicit any significant improvement in those parameters in both males and females. Gender was also significant factor influencing the effect of NaHCO_3_ on power in each second of the Wingate test and on performance in DT test. What is interesting, males significantly increased the number of throws in DT test, while females did not. That may suggest that the response to NaHCO_3_ treatment is gender specific.

As previously observed, supplementation with NaHCO_3_ may improve performance in combat sports^9–11^. NaHCO_3_ resulted in improvement of boxing (punch efficacy: +5.4%)^10^, taekwondo (the total attack time in combat: +13%)^11^ and judo (summed number of throws in three bouts of SJFT: +4 throws)^9^ specific performance, respectively. In contrast, in our study performance in wrestling-specific DT improved significantly on NaHCO_3_ with significant gender interaction. Then, when analysing genders separately we found that males increased the number of throws by ~11% (~2 throws), while no significant changes were observed in females. This slight, yet important change could contribute to winning in real wrestling competition. Previous studies on NaHCO_3_ in combat sports did not include female athletes^9–12^.

Gender differences in response to NaHCO_3_ supplementation are especially worth discussing. Papers with female subjects are scarce. Only one of six studies on women showed the improvement after NaHCO_3_ intake^21–26^. Kozak-Collins *et al*.^21^ supplemented seven competitive female cyclists with either 300 mg∙kg^−1^ NaHCO_3_ or PLA (NaCl). 2 h after ingestion participants performed interval cycling protocol consisting of repeating intervals of 1 min 95%VO_2_max cycling and 1 min recovery at 60 W until exhaustion. They did not find any improvement in the number of completed intervals. In comparison, Price *et al*.^27^ recruited only male subjects. Investigators also gave them NaHCO_3_ or PLA (NaCl) before testing. Participants did two intermittent cycling trials comprised of repeated 3-min blocks (90 s at 40%VO_2_max, 60 s at 60%VO_2_max, 14-s maximal sprint, 16-s rest). Authors found that compared to PLA, power output was greater throughout exercise during the NaHCO_3_ trial. Tiryaki and Atterbom^22^ assessed the effect of NaHCO_3_ on 600 m running time of trained females and found no differences (121.5 s on NaHCO_3_ and 120.4 s on PLA). On the other hand, males improved running time in 400 m distance by 1.52 s on NaHCO_3_ and in 800 m by 2.9s^28,29^. Even though there are no studies assessing the effect of NaHCO_3_ on 600 m run in males, it can be expected that it would be also improved. Then, there are four studies on female team sports players^23–26^. Macutkiewicz and Sunderland^23^ observed no influence of NaHCO_3_ on Field Hockey Skill Tests and the Loughborough Intermittent Shuttle Test in elite female field hockey players. In comparison, Krustrup *et al*.^30^ found 14% improvement in Yo-Yo intermittent recovery test level 2 performance (735 ± 61 m on NaHCO_3_ vs 646 ± 46 m on PLA) in trained males. Moreover, Ducker *et al*.^31^ and Miller *et al*.^32^ observed improved repeated sprint capacity in males on NaHCO_3_. In a study by Ducker *et al*.^31^ subjects did three sets of 6 × 20 m sprints with 4 min of recovery between sets. NaHCO_3_ resulted in the best repeated-sprint performance. In a study by Miller *et al*.^32^ male athletes were given NaHCO_3_ or PLA and then performed repeated sprint cycling protocol comprising 10 × 6 s sprints with 60 s recovery. Total work completed during the repeated sprint protocol was higher in the NaHCO_3_ condition (69.8 ± 11.7 kJ) compared with both - the control (59.6 ± 12.2 kJ) and PLA (63.0 ± 8.3 kJ) conditions. In a study on female team sports athletes NaHCO_3_ failed to improve total work in prolonged intermittent sprint performance (IST)^24^. IST consisted of two 36-min “halves” of repeated ~2-min blocks: all-out 4-s sprint, 100 s of active recovery at 35%VO_2_max, and 20 s of rest. There was a trend toward improved total work in the second half, but it did not reach statistical significance (*p* = 0.08). Similarly, no improvement was observed in female water-polo players^25^. After the ingestion of NaHCO_3_ or PLA the subjects performed a 59-min match-simulation test (MST) that included 56 ×10 m maximal-sprint swims. NaHCO_3_ increased blood pH, but failed to improve mean sprint times. The only study to show improvement on NaHCO_3_ in female athletes is a study by Delextrat *et al*.^26.^ Participants in that study were university basketball players. The supplementation protocol differed from all other studies. Athletes were supplemented with higher dose of NaHCO_3_ (0.4 g∙kg^−1^ compared to 0.3 g∙kg^−1^) and it was a multiday (3 days) loading. NaHCO_3_ improved mean values of sprint times, circuit times and jump height compared with PLA.

In summary, out of six trials on female athletes only in one NaHCO_3_ was proven to be effective^21–26^. On the other hand, males seem to benefit more from the supplementation^27–32^. The reason for that might be in physiological differences. Females have smaller type II fibers than men^17,18^. Type II fibers rely predominantly on glycolytic energy system. It was shown that males have greater glycolytic capacity^19,20^. In addition, in females pH drops to a lesser extent that in males during the same type of exercise^20^. All of that would explain the gender differences in the response to NaHCO_3_ supplementation observed in our and all previous studies.

Furthermore, it is important to observe that several bouts of intense exercise cause muscular fatigue, which may hamper performance during competition or training. In our study, power characteristics in WT_1_ tended to be higher than in WT_2_ (Table 1). One of the factors contributing to fatigue is a decrease in intramuscular pH, which causes reduction in enzyme activation, competitive binding of H^+^ to the active site of troponin, inhibition of oxidative phosphorylation and compromised resynthesis of phosphocreatine^6^. Sodium bicarbonate supplementation results in better buffering capacity of blood, which may increase the efflux of H^+^ and La^−^ out of muscle cells and decrease acidosis^4^.

It was previously established that the effect of NaHCO_3_ supplementation may be pronounced predominantly in latter stages of exercise^9,13–15^. Artioli *et al*.^9^ supplemented their athletes with 300 mg·kg^−1^ NaHCO_3_ 2 h before exercise. The performance test included four bouts of 30-s upper body WT tests. The significant changes in AP and PP were observed only in the two final bouts. This was attributed to improved resynthesis of phosphocreatine due to alkalosis caused by NaHCO_3_ supplementation, since low intramuscular pH may hamper this process^9^.

Tobias *et al*.^14^ assessed the effect of one week NaHCO_3_ ingestion on four-bout upper-body WT performance. Single bout was 30 s long with the load of 5% body mass. Seven-day supplementation resulted in 8% increase in total work done (in all four bouts summed). However, when the bouts were analysed separately a significant increase in AP and PP was present only in the last bout (+9.4%, *p* = 0.038, and +13.7%, *p* = 0.018, respectively)^14^.

A subsequent study by Oliveira *et al*.^15^ confirmed those results. They adopted a similar protocol of performance testing (four 30-s WT bouts for upper body interspersed by 3-min recovery) and also observed a significant increase in the total work done (+2.86%, *p* = 0.02) after 5-day NaHCO_3_ supplementation compared to PLA. And again the difference was more pronounced in the last two bouts (sum of bout 3 and 4: +5.93%, *p* = 0.02).

Since aforementioned studies^9,14,15^ showed that the effect of NaHCO_3_ is apparent the most in latter stages of intense exercise, we aimed at assessing the gender-related effect of NaHCO_3_ on the difference between the first and the second WT, which were additionally interspersed by DT. Dummy throw test is a highly strenuous test, specific to wrestling. It is comprised of two alternating modes – slow and fast^13^. The slow mode lasts 30 s, during which an athlete does four compulsory dummy throws. Whereas, in the quick mode an athlete performs as many throws as possible in 15 s. The test lasts 3 min and comprises four slow and four quick modes, so that it is highly exhausting. Thus, the participants of our study were already fatigued on the onset of the second WT. Even though the difference in PP between WT_2_ and WT_1_ tended to be improved by NaHCO_3_ (by 35.1 W and 35.0 W in females and males, respectively), they were not statistically significant (Table 2).

Furthermore, innovative analysing (in the field of NaHCO_3_ supplementation) of each second of WTs separately significant improvement (NaHCO_3post_ vs NaHCO_3pre_) in the difference in power between WT_2_ and WT_1_ were observed in seconds 12^th^, 16^th^ and 21^st^ when all participants were taken together. In females the significant difference was apparent only in 21^st^s (NaHCO_3post_ vs NaHCO_3pre_), whereas in males in 12^th^s (NaHCO_3post_ vs NaHCO_3pre_). Compared to PLA, on NaHCO_3_ the difference in power between WT_2_ and WT_1_ improved in seconds 12^th^, 16^th^, 17^th^ and 21^st^ in all participants. In females, significant improvement was observed in 21^st^s and in males in 17^th^s (NaHCO_3post_ vs PLA_post_). It therefore seems reasonable to emphasize that most of the substantial effects were observed in the case of this supplementation protocol in the middle (12–21s) of the WTs.

**Figure 4:**
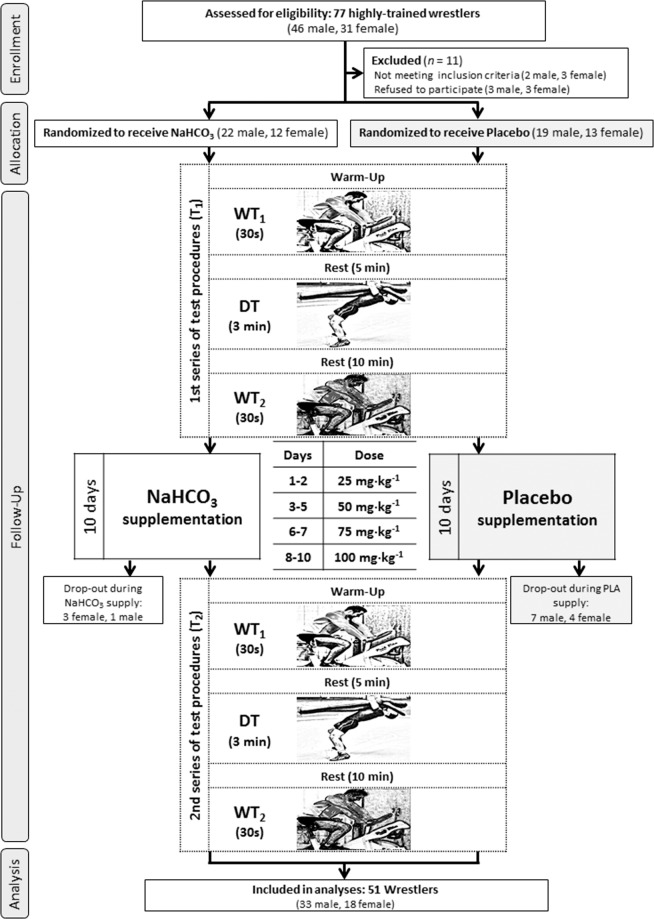
Flowchart of the study design.

In spite of the few significant differences observed in our study, we hypothesise that the dosage of NaHCO_3_ might have been too small for female and male wrestlers to elicit more apparent improvements. We used up to 100 mg·kg^−1^ NaHCO_3_ in days 8–10 of supplementation (Fig. 4). The dosage was well tolerated and did not cause any gastrointestinal (GI) problems, but the effectiveness was slight and moderate. Simultaneously, in previous studies higher doses were usually implemented^9,14,15,33^. IOC recommends the intake of 200–400 mg·kg^−1^ NaHCO_3_ 60–150 min prior to exercise^3^. However, in many athletes these doses result in GI distress^34^. This may prevent the practical use of supplementation with this compound in the conditions of natural high-intensity effort that is carried out, e.g. in combat sports. On the other hand, smaller doses might be ineffective. For instance, in nine healthy males the dose of 100 mg·kg^−1^ failed to induce alkalosis, increase base excess and had no influence on work output^35^. Furthermore, in six males McKenzie *et al*.^36^ showed that even though induced alkalosis was greater with 300 than 150 mg·kg^−1^ NaHCO_3_, there were no differences in work produced (133.5 and 133.1 kJ, respectively) and time to fatigue in the last bout (106 and 110s) between those two doses. However, comparing all those results to ours is limited because all of them used acute supplementation protocol, while participants in our study ingested NaHCO_3_ for ten days. In a previous study done by our lab, progressive-dose protocol of NaHCO_3_ up to 150 mg·kg^−1^ was enough to improve CrossFit-like performance and ventilatory threshold^37^. However, NaHCO_3_ supplementation protocol similar to the one used in the current study (10 days, up to 100 mg·kg^−1^) improved only time to PP in the second WT test with no further influence on anaerobic capacity and performance^13^. Nevertheless, we would like to highlight that in our research only highly-trained female and male wrestlers participated. Therefore, the observed changes related to males wrestling-specific performance and more effective maintenance of anaerobic power during high-intensity efforts, that can be considered beneficial at elite sport level, especially considering the short time duration of supplementation and a low dose of NaHCO_3_. It is worth bearing in mind, however, that a certain limitation to our study is the lack of verification of the bicarbonates concentration in the blood, which should be included in the subsequent studies, preferably in connection with the attempt to evaluate the effectiveness of supplementation of various doses of NaHCO_3_. Another limitation is the uneven distribution of participants in study groups. It is possible that if the number of participants was equal in each group the gender differences would be more pronounced.

## Conclusions

Progressive low-dose NaHCO_3_ supplementation allows in combat sports the advantageous suppression of fatigue-induced power decline in the midsection of the 30-s Wingate test and improvement in wrestling specific dummy throw test. The response to NaHCO_3_ supplementation seems to be gender dependent. It appears that males can benefit more from the sodium bicarbonate supplementation, possibly because of physiological differences.

## Methods

We would like to clarify that in this work we used the data previously collected in sodium bicarbonate studies involving wrestlers, which we conducted in our lab. We have already partially published the selected results obtained from most of the evaluated participants^13^. However, the data presented here was analyzed in a completely different fashion. The results for female and male wrestlers were analyzed separately to assess whether the response to the supplementation protocol may be determined by the gender of the athletes. We also focused on previously untouched aspects of the detailed change of power indices during each seconds of the Wingate test. Additional athletes were also included. Thus, we can unequivocally state that there is absolutely no risk of duplicate results, but we want to inform potential readers about the details of the data processing. Lastly, from a practical and scientific perspective, the research results which we have presented here are extremely valuable due to the innovative approach we have taken with NaHCO_3_ supplementation, the detailed analysis of performance indices herein, and the accompanying assessment of gender-related responses to NaHCO_3_ treatment.

### Participants

Forty-six male and thirty-one female wrestlers were initially enrolled in this study. However, thirty-three male and eighteen female athletes participated in the study and were included in the analyses (Fig. 4). Anthropometric characteristics are presented in Table 4. The athletes were members of the Polish Wrestling National Team and/or competed in the highest level of Polish competitions. The inclusion criteria were good health, a valid medical clearance to participate in sports, a minimum of four years of combat sports experience, and doing at least four workout sessions (combat sport) a week. The exclusion criteria were current injury, any health condition preventing from participation, self-declared unwellness, and no interest in proper participation in study protocol.

**Table 4:** Anthropometric characteristics of female and male wrestlers.

	Females		Males	
	NaHCO_3_ (n = 9)	PLA (n = 9)	NaHCO_3_ (n = 21)	PLA (n = 12)
Age (yrs)	18.7 ± 2.4	18.1 ± 2.6	19.7 ± 3.8	19.5 ± 4.4
Body height (cm)	165 ± 7	169 ± 6	176 ± 8	174 ± 7
Body mass (kg)	53.7 ± 5.1	64.5 ± 8.1	78.8 ± 13.1	75.3 ± 13.3
FM (%)	17.3 ± 3.0	19.1 ± 4.6	11.5 ± 4.2	10.5 ± 2.8
FM (kg)	9.4 ± 2.5	12.5 ± 4.3	9.4 ± 4.8	8.0 ± 3.6
FFM (%)	81.6 ± 3.8	82.1 ± 6.4	88.5 ± 4.2	89.7 ± 3.0
FFM (kg)	44.3 ± 3.1	52.0 ± 4.8	69.2 ± 9.5	67.3 ± 10.6
TBW (%)	59.1 ± 2.9	55.9 ± 3.6	62.0 ± 5.4	63.0 ± 3.5
TBW (kg)	31.6 ± 1.5	35.8 ± 3.0	48.7 ± 6.3	46.9 ± 7.2

Data are presented at mean ± standard deviation (SD). FM, fat mass; FFM, fat-free mass; TBW, total body water content.

All athletes reassured that they had not changed their life-styles, training regimen, diet or supplementation, and that they had not been using any medications and supplements with potential ergogenic effects, other than those supplied by the authors of this study. In accordance with the 1975 Declaration of Helsinki, before enrolment all participants had given their written consents to participate in the study protocol. Informed consents were also obtained from the parents of athletes under the age of 18 years, prior to participation in the study. The approval of the Bioethics Committee at Poznan University of Medical Sciences was obtained for this study. This trial was registered at Clinical Trials Gov (website: https://clinicaltrials.gov/ct2/show/NCT03406065; Clinical Trial Identification Number: NCT03406065). The study was registered retrospectively as registration was not required when the study enrolment started. The authors confirm that all ongoing and related trials associated with this intervention are registered. The study complies with the CONSORT statement for randomized trials, as shown in Fig. 4.

### Study design and protocol

The study was designed as randomized double-blind placebo-controlled parallel-group trail. The supplementation period lasted ten days. The participants were familiarized with the exercise testing protocol and the equipment on a preliminary meeting with the research team. Anthropometric measurements were also taken on the same day. When enrolled athletes were randomly divided into the treatment groups (the NaHCO_3_ group or the PLA group). The random allocation sequence and matching were performed using stratified randomization via impartial biostatistics.

The experiment consisted of two separate visits (T_1_–T_2_) for each participant. All testing was performed in natural conditions at the Central Olimpic Sports Centers (Spała, Zakopane) and Wrestling Training Centers (Poznań) in Poland. Throughout the study the participants were supplemented with either NaHCO_3_ or PLA. Exercise tests were conducted before and after each trial at the same time of day. Testing sessions started between 7.30 and 10.00 a.m. each time. To maintain constant conditions the participants were asked to refrain from any strenuous exercise for 24h before the testing.

### Supplementation

The participants were supplemented with NaHCO_3_ for ten consecutive days. Initial dose was much smaller than the dose recommended previously^2,3^ and was then increased gradually until 0.1 g∙kg^−1^ was reached. This loading protocol was shown to eliminate any GI side effects^13,37^. Supplementation protocol is depicted in Fig. 4. Sodium bicarbonate was administered in the form of unmarked disc-shaped tablets (Alkala T, manufacturer–Sanum Kehlbeck GmbH & Co. KG, Germany). The tablets were ingested with at least 250 mL of water and could either be swallowed or dissolved in the mouth. Maltodextrin with NaCl served as PLA. It was administered in a similar tablet prepared by the same producer as of the NaHCO_3_ tablets.

Daily doses of both NaHCO_3_ and PLA were split into three even portions. On training days, the tablets were ingested in the morning, in the evening, and 1.5h before training session. On rest days, the supplements were administered in the morning, in the afternoon, and in the evening. To increase adherence the participants were also given personal supplementation plans.

### Anthropometric measurements

Anthropometric measurements were taken in the fasted state in the preliminary visit in the morning. Body fat and free-fat mass were assessed based on air displacement plethysmography using the Bod Pod® (Cosmed, Italy)^38^. Total body water and hydration level were assessed by means of bioelectric impedance, with Bodystat 1500 (Bodystat Inc., UK)^39^, and via urine specific gravity measurement, with URYXXON® Relax (Macherey-Nagel, Germany).

### Exercise tests

Every testing session consisted of two Wingate anaerobic tests interspersed with a dummy throw test. Wrestling-specific performance capacity was measured using a specific dummy throw test described previously^13^. Wingate tests were performed on a cycloergometer (Monark 894E, Sweden). All recommendations for such tests as proposed by Bar-Or were strictly followed^40^. External loading was set at 7.5% body weight. The first WT (WT_1_) was performed 5 min before DT and the second (WT_2_) 10 min after DT (Fig. 4). Prior to testing all athletes completed 5-min warm-up on cycloergometer at approximately 50 W power. During the test, the athletes were verbally encouraged to exert maximum effort. The recorded results were analysed using the Monark Anaerobic Test Software (ver. 3.0.1, 2009, Monark, Sweden).

### Blood samples analysis

Fingertip blood samples were taken twice, one sample before the WT_1_ and the other 3 min after the WT_2_. During blood draws the participants seated in an upright position. Blood samples were immediately transferred to microtubes containing 500 µL of 0.6 M perchloric acid. Glucose concentration was measured using a colorimetric enzymatic method with glucose oxidase (Liquick Cor-GLUCOSE, Cormay, Łomianki, Poland). Lactate and pyruvate measurements were performed according to the method described previously^13^. All biochemical analyses were conducted using a Synergy 2 SIAFRT microplate multi-detection reader (BioTek, USA).

### Statistical analysis

The study was designed as a randomized parallel trial. Thus, in statistical analysis a mixed model of repeated measures with known error covariance matrix was used^41,42^. The random factor was participants nested in groups. Group stand for treatment (NaHCO_3_ or PLA). Fixed factors were: period (NaHCO_3pre-WT1_, NaHCO_3pre-WT2_, NaHCO_3post-WT1_, NaHCO_3post-WT2_, PLA_pre-WT1_, PLA_pre-WT2_, PLA_post-WT1_, PLA_post-WT2_), gender, times (period) (1–30 seconds of WT). Two-way interactions (gender × treatment, treatment × period, gender × period, and treatment × times (period)) and three-way interactions (gender × treatment × period, and gender × treatment × times (period)) were considered. Tested error covariance matrix structures included: Compound symmetry, Autocorrelation, Toeplitz and Unstructured. The choice of model with adequate covariance matrix structure was done according to Akaike information criterion^43^. Because gender and gender interactions with other factors were usually significant, those analyses were performed also for both genders separately. Statistical significance was set at *p* < 0.05. The assumptions of normality and homoscedasticity was tested using the Shapiro-Wilk test for normality. If data did not meet the assumptions then the Box-Cox transformation was used. Data were analyzed by own calculations and using the SAS 9.3 software program. Effect size was calculated as Cohen’s ƒ2, as follows: f 2 = h 2 /(1 – h 2).

### Ethical approval

All procedures performed were in accordance with the ethical standards of the institutional and national research committee and with the 1975 Helsinki declaration and its later amendments or comparable ethical standards.

## Practical Applications

Our study suggests that 10-day progressive low-dose (from 0.025 g·kg^−1^ (days: 1–2) up to 0.1 g·kg^−1^ (days: 8–10)) NaHCO_3_ supplementation allows the advantageous strengthening of wrestling-specific performance in males and suppression of fatigue-induced average power decline in combat sports, which is a result of specific physical efforts. It can also lead to maintenance of high anaerobic power mainly in the midsection of the 30-second Wingate test. Moreover, the higher dose could be more effective in this respect, which indicates that despite the lack of effect on GI functioning, doses lower than 0.1 g·kg^−1^ BM do not seem to be effective in combat sports. It seems, however, that the response to NaHCO_3_ supplementation may be gender dependent, and males could be more prone to sodium bicarbonate supplementation.

## Data Availability

The datasets generated during and/or analysed during the current study are available in the figshare database repository (https://figshare.com/s/cf05c5daeb7e4b4f310e; 10.6084/m9.figshare.7907879).
